# Synthesis of Backbone-Modified Morpholino Oligonucleotides Using Phosphoramidite Chemistry

**DOI:** 10.3390/molecules28145380

**Published:** 2023-07-13

**Authors:** Sibasish Paul, Marvin H. Caruthers

**Affiliations:** 1 Nucleic Acid Solutions Division, Agilent Technologies, Boulder, CO 80301, USA; 2 Department of Biochemistry, University of Colorado, Boulder, CO 80303, USA

**Keywords:** phosphorodiamidate morpholinos (PMO), phosphoroamidate morpholinos (MO), thiophosphoramidate morpholinos (TMO), phosphoramidites

## Abstract

Phosphorodiamidate morpholinos (PMOs) are known as premier gene knockdown tools in developmental biology. PMOs are usually 25 nucleo-base-long morpholino subunits with a neutral phosphorodiamidate linkage. PMOs work via a steric blocking mechanism and are stable towards nucleases’ inside cells. PMOs are usually synthesized using phosphoramidate P(V) chemistry. In this review, we will discuss the synthesis of PMOs, phosphoroamidate morpholinos (MO), and thiophosphoramidate morpholinos (TMO).

## 1. Introduction

**Figure 1:**
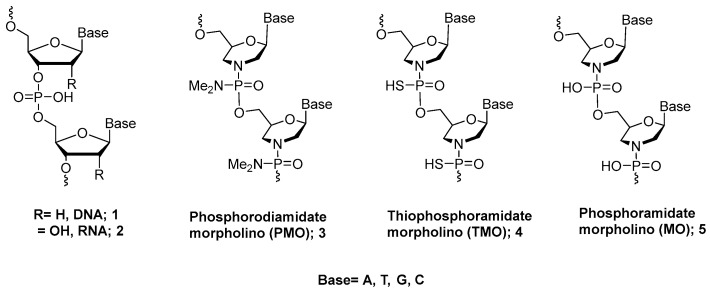
Chemical structure of morpholino oligonucleotides.

Among the various oligonucleotide analogues that have been used as therapeutic drugs, the phosphorodiamidate morpholino oligonucleotide (PMO; **3**) remains one of the most successful (Figure 1). Of the 16 oligonucleotide therapeutics approved so far by the US Food and Drug Administration (FDA), 4 are PMO-based drugs. These are Eteplirsen (2016), Golodirsen (2019), Viltolarsen (2020), and Casimersen (2021) for the treatment of Duchenne Muscular Dystrophy (DMD) [1,2,3,4]. Eteplirsen and Golodirsen are used for the treatment of DMD, targeting exons 51 and 53, respectively, of the dystrophin mRNA. The other dystrophin drugs are Viltolarsen and Casimersen, which target exons 53 and 45, respectively. DMD is a rare genetic disease of the dystrophin protein and predominantly affects males. In healthy muscle, dystrophin interacts with other proteins at the cell membrane to stabilize and protect the cell during regular activity involving muscle contraction and relaxation. Individuals with DMD produce little or no dystrophin in their muscle. Without dystrophin, normal activity causes excessive damage to muscle cells and over time is replaced with fat and fibrotic tissue. PMOs bind to a target out-of-frame dystrophin pre-mRNA and alter splicing, which results in an in-frame mRNA. Consequently, a truncated but functional dystrophin protein is produced.

The basic PMO structure consists of the six-membered morpholine ring, instead of ribose, attached to the same nucleobases as RNA [5]. PMOs possess a neutral backbone that is completely resistant to nucleases. They are water soluble and display good cell permeability without assistance from cellular factors. They also hybridize to RNA but do not activate RNase H [6,7].

**Scheme 1:**
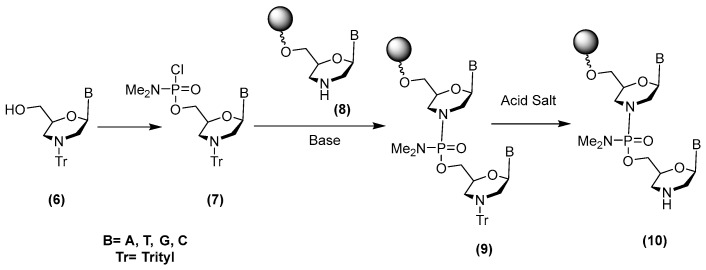
Method for synthesis of PMOs using chlorophosphoramidate chemistry.

PMOs are currently synthesized with phosphoramidate P(V) chemistry on a solid support using chlorophosphoramidate building blocks, as described in Scheme 1. The first step is condensing a 6′-hydroxyl-N-tritylmorpholino nucleoside **6** with N,N-dimethylaminodichlorophosphoramidate in order to generate an N,N-dimethylamino chlorophosphoramidate synthon **7**. Coupling the base of this synthon to a morpholino nucleoside linked to a support through the 6′-hydroxyl **8** generates the dimer **9** attached to the resin. Further detritylation with an acid salt yields a product **10** that can be elongated by repeating the cycle [7]. Several limitations of this synthesis approach have been reported [8]. PMOs are synthesized in a 5′ to 3′ direction, whereas almost all other oligonucleotide synthesis chemistries take place in a 3′ to 5′ direction [9]. Thus, the synthesis strategy for PMOs is not compatible with most other chemistries, which severely limits the ability to synthesize chimeras having PMOs and other analogues. Additionally, the active chlorophosphoramidate synthon is a P(V) compound which is less reactive and therefore leads to significantly lower yields than the P(III) oligonucleotide synthesis strategy.

Because of the versatility of P(III) chemistry, several DNA/RNA derivatives in addition to natural DNA and RNA can be synthesized efficiently [10]. Thus, to develop an orthogonal synthetic approach for PMOs, we have explored the use of P(III) chemistry for preparing the PMO analogue and its derivatives on a solid support. If successful, a P(III) strategy would lead to procedures for preparing many oligonucleotide derivatives. In this short review, we will discuss the synthesis of PMOs and additional PMO-type analogues (MOs and TMOs, Figure 1) using phosphoramidite P(III) chemistry. Also, the biological properties of the newly developed TMOs will be discussed.

## 2. Initial Work

**Scheme 2:**
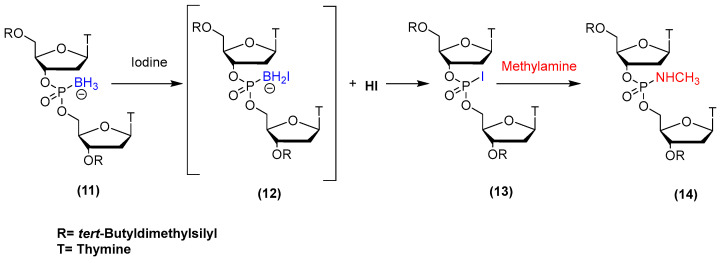
Synthesis scheme for phosphoramidate DNA from boranephosphonate DNA.

In 2015, we published our research regarding the conversion of boranephosphonate DNA to phosphoramidate DNA [11], as outlined in Scheme 2. The approach first involved the conversion of an internucleotide phosphite linkage to boranephosphonate using the BH_3_-THF complex in THF to yield **11**. This condensation was followed with 30 equivalents of iodine and a 0.4 M solution of methylamine in tetrahydrofuran, which led to the formation of methylphosphoramidate **14**. This breakthrough chemistry enabled us to synthesize phorphoramidate oligodeoxyribonucleotides starting from boranephosphonate DNA. Of particular interest was the observation that a diastereomerically pure borane phosphonate isomer gave rise to the formation of one diastereomer via a stereospecific reaction [11]. This work encouraged us to attempt the synthesis of PMOs using the same iodine oxidation chemistry.

## 3. Synthesis of PMOs and PMO/DNA Chimeras

**Figure 2:**
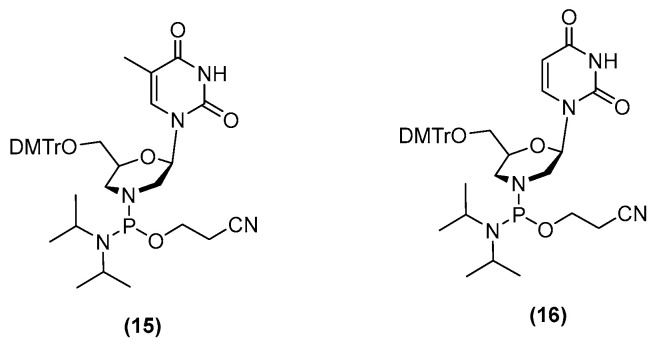
Phosphorodiamidite morpholino building blocks.

Zhang and collaborators reported the synthesis of chimeric oligonucleotides having 2′-deoxyribonucleoside-3′-phosphate and morpholinonucleoside 3′-phosphoramidate internucleotide linkages [12,13]. They used morpholino uridine and thymidine phosphorodiamidites (Figure 2) to synthesize these oligonucleotides at a 0.1 µmol scale on a DNA synthesizer using phosphoramidite chemistry and 5-ethylthio-1*H*-tetrazole (ETT) as the activator. Additionally, oligonucleotides with morpholino phosphoramidate modifications in the 3′-terminal ends were synthesized using the universal CPG support. Aqueous ammonia at 55 °C for 12 h was then used to separate the oligonucleotides from the support and to remove base protecting groups. Crude oligonucleotides were purified using HPLC with yields of approximately 30% and analyzed via HPLC and MALDI-TOF mass spectroscopy. Using this approach, siRNAs containing up to four morpholino uridine phosphoroamidate linkages were synthesized. Although several interesting biological properties were reported (resistance to nuclease, silencing activity in HeLa cells) no further research has been reported. These results encouraged us to explore the synthesis of PMOs on solid supports using the P(III) chemistry we had developed previously for the synthesis of the corresponding 2′-deoxyoligonucleotide analogues [11].

**Scheme 3:**
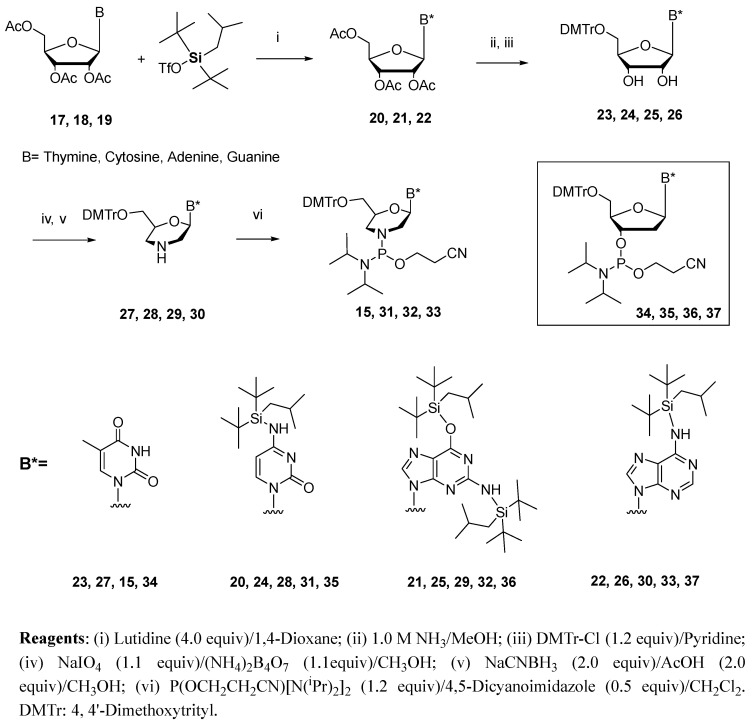
Synthesis of phosphorodiamidite building blocks for the preparation of PMOs.

Our first step toward the synthesis of PMOs using a P(III) phosphoramidite approach was to synthesize all four morpholino phosphorodiamidite building blocks (Scheme 3). Our overall strategy was to use these building blocks for the preparation of morpholino borane phosphonate derivates and then convert these linkages to PMOs using iodine oxidation [14,15]. However, the standard amide protecting groups routinely used for oligonucleotide synthesis cannot be used with cytosine, adenine, and guanine because boronation reduces these amides to amines, which consequently cannot be removed following synthesis. Therefore, we turned to the previously developed bis(tert-butyl)isobutylsilyl (BIBS) group [11] for protection of the amino groups on these three bases because the BIBS protecting group was stable to P(III) chemistry synthesis procedures, including boronation, and could be readily removed with fluoride.

The preparations of these four building blocks, as outlined in Scheme 3, were based upon modifications of the originally published procedures [16,17]. 5′-O-Dimethoxytrityl ribothymidine **23** was treated with sodium periodate and then ammonium biborate to afford the dihydroxythymine morpholino monomer. Reduction using sodium cyanoborohydride under mild acidic conditions yields the thymine morpholino monomer **27** [17]. The morpholino–thymine synthon **15** was then prepared in an 86% yield via the phosphitylation of **27** with 2-cyanoethyl-N,N,N′,N′-tetraisopropylphosphordiamidite and 4,5-dicyanoimidazole (DCI) in dichloromethane under an argon atmosphere [12]. Synthesis of the cytosine, adenine, and guanine monomers began by first protecting the nucleoside amino groups with BIBS. The reaction of 5′,3′,2′-tri-O-acetylcytidine **17** with BIBS-OTf (Tf = triflate) in the presence of 2,6-lutidine gave a 76% yield of **20** after 2 h of stirring at 60 °C under argon. The synthesis of **21** and **22** from **18** and **19**, respectively, required much longer reaction times (3 days), with yields of 25−31% and 74%. These silylated ribonucleosides were then treated with ammonium hydroxide to remove the acetyl protecting groups, converted them to the 5′-dimethoxytrityl compounds and then to the morpholino derivatives (**28**, **29**, and **30**) using the same chemistry as outlined for the preparation of **27**. These compounds were used to generate the phosphorodiamidite building blocks **31**, **32**, and **33**. For the synthesis of PMO−DNA chimeras, 5′-O-(4,4′-dimethoxytrityl)-2′-deoxyribonucleoside-3′-phosphoramidite building blocks **35**, **36,** and **37** were prepared following a literature protocol [14,15], whereas compound **34** was obtained from commercially available sources.

**Scheme 4:**
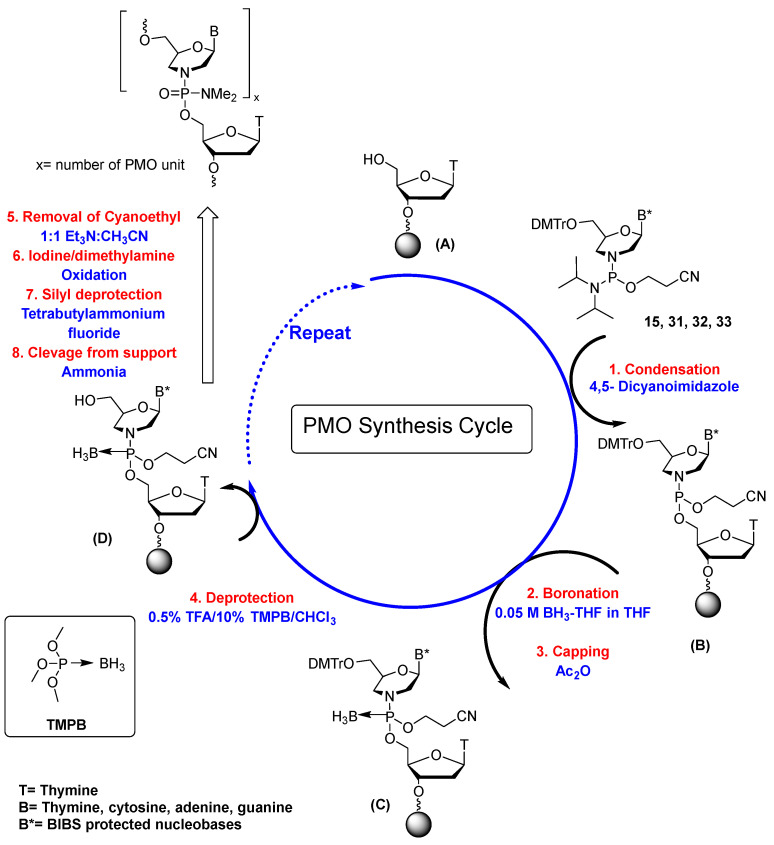
Synthesis cycle for the preparation of PMOs.

The synthesis cycle for the preparation of PMOs using P(III) chemistry is outlined in Scheme 4 [15]. The synthesis begins with commercially available 5′-DMT-2′-deoxyribothymidine linked to a polystyrene support. The standard glass synthesis support (CPG) could not be used because this support was not compatible with fluoride reagents that were required to remove N-silyl ether protecting groups from the bases. Compound (**A**) can be any of the 2′-deoxyribonucleosides, but the cytosine, adenine, and guanine bases must be protected with the BIBS protecting group. The DMT group was removed with 0.5% trifluoroacetic acid in chloroform containing 10% trimethyl phosphite–borane (TMPB). To completely remove this detritylation solution, it was important to use a methanol wash [14]. The 5′-unprotected-2′-deoxyribonucleoside (**A**) was then reacted with **15**, **31, 32**, or **33** in anhydrous acetonitrile containing 4,5-dicyanoimidazole (DCI) to generate a dimer having a phosphoramidite diester internucleotide linkage (**B**). Boronation was next carried out using borane–THF to generate a P (IV) morpholino compound (**C**) prior to the capping step. Post-boronation, the support was washed with acetonitrile, failure sequences were capped using acetic anhydride, and detritylation was carried out using a solution of 10% TMBP and 0.5% TFA in chloroform in order to generate (**D**). Following a methanol wash, multiple repetitions of this cycle yielded a product ready for further conversion to the PMOs (Table 1). Post-synthesis, supports were washed with acetonitrile, treated with a 1:1 mixture of triethylamine/acetonitrile to remove the cyanoethyl protecting group, washed with acetonitrile and dichloromethane to remove residual triethylamine, and dried. For conversion to the N,N-dimethylamino PMOs, the morpholino boranephosphoroamidates were treated for 2 h with a solution of 0.05 M iodine and 2.0 M dimethylamine in tetrahydrofuran. Using the procedure outlined in Scheme 4, a trimer having only thymine nucleoside bases was synthesized, converted to the N,N-dimethylamino PMO derivative with iodine/N,N-dimethylamine, removed from the support with ammonia, and analyzed by LC−MS. A 94% yield of the trimer PMO was calculated. PMOs having oligothymidine and all four bases have been synthesized using this approach and characterized by LC−MS.

For the synthesis of PMO−DNA chimeras, 5-ethylthio-1H-tetrazole (0.25 M and 180 s coupling time) was used as an activator for the 5′-O-(4,4′-dimethoxytrityl)-2′-deoxyribonucleoside-3′-phosphoramidites (**34, 35, 36**, or **37**). Following the addition of the 2′-deoxyribonucleotide, iodine oxidation converted the phosphite triester to the corresponding phosphate linkage. Since PMO−DNA chimeras are new to the scientific community and could prove to be useful for various research projects, several were synthesized. Initially, these chimeras were a series of 21mer oligothymidines containing four N,N-dimethylamino PMO linkages. These encouraging results were followed by the synthesis of PMO−DNA chimeras containing all four nucleobases with variable locations and a number of PMO linkages [15].

In addition to testing this new synthetic route by synthesizing PMO analogues having the N,N-dimethylamino–phosphorodiamidate linkage, we investigated other amines, which could be used in order to generate several new PMO−DNA derivatives. An oligothymidine 21mer having four morpholino boranephosphoroamidate linkages near the center of this oligomer was synthesized. The support containing this oligonucleotide was divided into three samples that were treated with N-methylamine, ammonia, and morpholine under iodine oxidation conditions and then purified using reverse-phase column chromatography. Additionally, mixed-sequence PMO−DNA chimers having all four bases and amino-phosphorodiamidate internucleotide linkages were synthesized, where the positions for the diamidate linkages were located at the 5′, 3′, and 5′/3′ termini of these chimeras. Yields were comparable to those obtained for the N,N-dimethylamino PMO chimeras [15].

## 4. Synthesis of MOs and MO/DNA Chimeras

As outlined earlier in this review, Zhang and collaborators [12,13] synthesized oligonucleotides having up to four thymidine morpholino phosphoramidate internucleotide linkages (MOs, **5** Figure 1). The biochemical results from these investigations were intriguing. These MOs formed duplexes with complementary RNA and were stable toward degradation with snake venom phosphodiesterase. Moreover, when up to four morpholino uridine phosphoramidates were incorporated into siRNA duplexes, the resulting RISC complex showed potent silencing activity in HeLa cells.

**Scheme 5:**
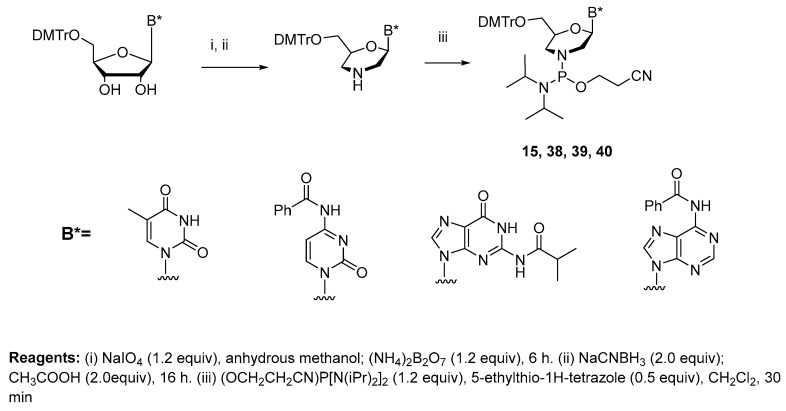
Synthesis of phosphorodiamidite morpholino building blocks for MOs and TMOs.

Because of these exciting results, MO synthesis was extended to all four standard bases, with these oligonucleotides having 15 to 20 nucleotides [18]. The building blocks used for this research are shown in Scheme 5. Because there is no boronation step, as was the case for the synthesis of PMOs via P(III) chemistry, the standard amide-protecting groups were observed to be appropriate for studies on MO synthesis (Scheme 5; **15**, **38**, **39**, **40**). Following an extensive study focused on the activation of these building blocks with 5-ethylthio-1H-tetrazole (ETT), a solid-phase synthesis cycle (Table 2) was developed for preparing MOs [18]. However, despite considerable experimentation, the MOs could not be isolated free of oligonucleotide degradation products. The problem primarily centers on the reactivity of the morpholino phosphoramidate linkage toward aqueous acid. Thus, during the final deprotection step using aqueous acid to remove the 5′-O-(4,4′-dimethoxytrityl) group, considerable degradation of the MO was observed, and the final MO product could not be isolated in pure form. Although the synthesis of MOs with all four bases is now possible, it is clear that a different synthesis strategy must be introduced in order to overcome the inherent instability of the morpholino phosphoramidate linkage toward aqueous acid. As stated in the referenced manuscript [18], “However, due to the inherent acid and base stability of P-N linkages in phosphoramidate ODNs, an improved synthetic methodology that integrates orthogonal protecting group strategies for the morpholino nucleoside building blocks as well as the terminal hydroxyl protecting group of the final ODN may be required to enable their successful isolation for further research.”

## 5. Synthesis of TMOs and TMO/DNA Chimeras

**Scheme 6:**
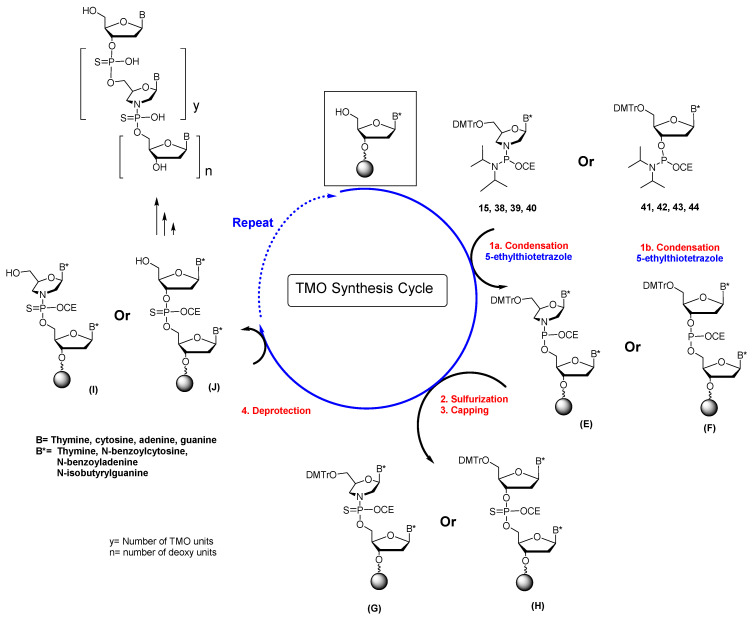
Synthesis cycle for TMOs and TMO-DNA chimeras.

The solid-phase synthesis of MOs was carried out by oxidizing compound B, as shown in Scheme 4. The resulting morpholino phosphoramidate oligonucleotides were obtained in low overall yields, and their crude reaction mixtures could not be purified using the conventional DMT-On/Off procedure due to their instability to aqueous acid. We conjectured that replacing the nonbridging P(O) linkages in morpholino phosphoramidates with P(S) might improve their hydrolytic stability under acidic conditions and increase nuclease resistance towards intracellular enzymes. This presumption led us to recently focus entirely on thiophosphoramidate morpholino oligonucleotides (TMOs, **4** Figure 1) [15,19]. Morpholino phosphorodiamidites **15, 38, 39**, and **40** (Scheme 5) were used as building blocks to prepare TMOs. Because we did not have a boronation step in the TMO synthesis strategy, the standard amide protecting groups were used for the exocyclic amino groups of cytosine, guanine, and adenine. Several activators (ETT; Tetrazole; Dicyanoimidazole (DCI)) at various concentrations were studied, with the best activation of morpholino phosphorodiamidites being ETT at 0.12 M. The first step for synthesizing TMOs was to remove the 5′-DMT group from the 2′-deoxynucleoside attached to the silica support (Scheme 6 and Table 3). This group was removed using 3% trichloroacetic acid in dichloromethane. This 2′-deoxynucleoside was then reacted with a morpholino phosphorodiamidites of mABz, mGiBu, mCBz, or mT (**15, 38, 39**, and **40**) in the presence of 0.12 M ETT in anhydrous acetonitrile (300 s condensation time). The resulting morpholino phosphoramidite (**E**) was then converted to the thiophosphoramidate dinucleotide (**G**) using 3-[(dimethylaminomethylene)amino]-3H-1,2,4-dithiazole-5-thione (DDTT). Subsequent detritylation followed by repetitive synthesis cycles generated a TMO oligonucleotide having the desired sequence and length. When using the Universal Support III in order to generate a TMO having only morpholino nucleosides, the first morpholino phosphorodiamidite coupling step was followed by oxidation instead of sulfurization. Thus, during the ammonia cleavage of the TMO from the support, a TMO would be generated having a 3′-morpholine.

The solid-phase synthesis of TMOs and their chimeras resembles the synthesis of phosphorothioate DNA synthesis, with two differences: (i) the ETT concentration was reduced to 0.12 M, and (ii) a 300 s coupling time was used. We noticed that reducing the coupling time of phosphorodiamidite building blocks (**41, 42, 43**, or **44**) to 300 s (0.12 M ETT) resulted in synthesis yields for 20−25 mer TMOs that were approximately 50%-60% the amount for a phosphorothioate DNA on the same support. For the synthesis of TMO−DNA chimeras, a 30 s coupling time was used for commercially available 2′-deoxyribonucleoside 3′-phosphoramidites.

The final steps for the synthesis of TMOs and TMO/DNA chimeras begin with cleavage from the synthesis support and removal of protecting groups using aqueous ammonia at 55 °C for 18 h (for the Universal Support III, 2 M ammonia/methanol, 60 min followed by aqueous ammonia, 55 °C, 16 h). Post-deprotection, the ammonia solutions were removed by evaporation. Product mixtures were dissolved in 3% aqueous methanol, filtered through a 0.2-micron filter, and analyzed via LCMS. The reaction mixtures were redissolved in 3% aqueous acetonitrile and purified using ion-pair RP-HPLC. Post-purification, selected fractions were dried and treated with 500 µL of 50% aqueous acetic acid to remove the DMT group (5 min). The acidic reaction mixture was cooled in an ice bath followed by neutralization with triethylamine. After evaporating to dryness, the TMOs and TMO/DNA chimeras were re-purified using ion-pair RP-HPLC. The pure product fractions were combined and evaporated to dryness. Desalting was carried out using NAP DNA purification columns.

We have successfully developed a chemical synthesis strategy for synthesizing TMOs and TMO/DNA chimeras using phosphoramidite chemistry. In contrast to P(V)-based PMO synthesis, the P(III) approach lends itself to the incorporation of several modifications, such as 2′-OMe, 2′-F, 2′-O-MOE, LNA, and others, that provide a route for generating an array of previously unexplored oligonucleotide therapeutic drug candidates.

## 6. Biological and Biochemical Properties of TMOs

Since the binding affinity of an oligonucleotide plays a critical role in determining its therapeutic potential, the biophysical properties of TMOs and TMO/DNA chimeras have been analyzed [19]. Chimeric TMOs with alternating thiophosphoramidatemorpholino/2′-deoxynucleoside 3′-thiophosphate structures showed an increase in binding affinity (+6−10 °C) toward DNA. This could be because of increased flexibility to an otherwise rigid construct, which permits more efficient binding with the complementary strand. A significant Tm loss (>12 °C) was observed for the fully modified thiophosphoramidatemorpholino/2′-deoxynucleoside 3′-thiophosphate oligomer when forming a duplex with complementary DNA. This result suggests that the exclusive presence of rigid morpholino moieties results in a backbone conformation that is not helpful to efficient DNA binding. However, when TMOs form duplexes with complementary RNA, both the chimeric TMO/DNA and the completely thiomorpholino oligonucleotides form duplexes where the Tms are 10 degrees higher than for the RNA/DNA duplex. To gain further insights into their helical structure, RNA heteroduplexes of TMOs with complementary RNA were assessed using circular dichroism (CD) and compared with control duplexes (DNA−pS/RNA and canonical DNA/RNA). The CD spectra of these TMO heteroduplexes revealed structural similarities to the DNA−pS/RNA duplex. Both fully modified TMO/RNA and chimeric TMO/RNA duplexes closely resemble an A-form RNA/RNA duplex which provides a logical explanation for their higher binding affinity towards complementary RNA. The RNase H1 activity of TMOs was assessed by preannealing TMOs with complementary RNA and treatment with Escherichia coli RNase H1. Fully modified TMOs do not activate RNase H1, presumably due to their conformational rigidity. However, gapmers having TMO caps and 2′-deoxynucleoside 3′-thiophosphate gaps are RNase H1 active. This suggests that exclusively TMO-modified oligonucleotides are potential candidates for exon skipping experiments, where they function as steric blockers, whereas gapmers having TMO wings can be used for antisense experiments that involve mRNA degradation through the activation of the RNase H1 pathway.

TMOs were used to address how intron retention influences the spatio-temporal dynamics of transcripts from two clinically relevant genes: TERT (Telomerase Reverse Transcriptase) pre-mRNA and TUG1 (Taurine-Upregulated Gene 1) lncRNA [20]. Data suggest that the splicing of TERT-retained introns occurs during mitosis. In contrast, TUG1 has a bimodal distribution of fully spliced cytoplasmic and intron-retained nuclear transcripts. Upon the addition of TMOs complementary to TERT and TUG 1 preRNA, nuclear TERT and TUG1 intron retentions are correlative and do not show a causality of intron retention driving their subcellular localization. To test this hypothesis, 20mer TMOs were designed against the two TUG1 donor splice sites, each hybridizing to 2 nt of the exon and 18 nt of the intron sequence. These TMOs were tested for the functionality of intron-retention events using RNA targeting to block intron excision. It was observed that TMOs enforcing intron 1 retention decreased the splicing of intron 1 by ~60% compared to the control TMO. Intron 11-containing TERT (assessed by monitoring intron 11 and exon 11 to intron 11 junction) was increased ~32% compared to control TMO. As additional controls, we applied primers at the upstream exon 10 to exon 11 junction, which was not affected with TERT TMO treatment, and exon 10 to exon 12 junction, which was decreased ~50%, in accordance with the decrease in exon 11 to exon 12 junction. These findings demonstrate that TMOs effectively block splicing and change cellular localization and availability of the RNA.

In another study, various TMOs were synthesized and evaluated for their efficacy to induce exon skipping in a Duchenne Muscular Dystrophy (DMD) in vitro model using H2K mdx mouse myotubes [21,22]. Experiments demonstrated that TMOs can efficiently internalize and induce excellent exon-23 skipping potency compared with a conventional PMO control and other widely used nucleotide analogs, such as 2′-O-methyl and 2′-O-methoxyethyl antisense oligonucleotides. TMOs performed well at low concentrations (5–20 nM); hence, the dosages can be minimized, which may improve the drug safety profile. These results in the H2K mdx myotubes demonstrate an opportunity for using TMOs as therapeutic ASOs in the treatment of various genetic diseases.

These experiments strongly support the conclusion that TMO oligonucleotides are quite active in cell nuclei. This is because the excision of introns is a nuclear activity.

## 7. Conclusions

Chemical methods, focused on the use of nucleotide P(III) building blocks, are used for the synthesis of oligonucleotides that have found major transformative applications in biology, biochemistry, molecular biology, and biophysical chemistry. These building blocks are extremely stable, easily activated, and readily prepared. Recently, we have used P(III) chemistry to prepare several morpholino phosphorodiamidite building blocks to synthesize many novel PMO and TMO analogues. Currently, more than 20 biological studies focused on the use of TMOs as therapeutic drugs for the treatment of various genetic diseases are underway. These collaborations are being carried out with cells in culture, and several have progressed to studies in mouse models. Without exception, TMOs are more active than any other analogue tested. However, major obstacles such as challenging large-scale production and extensive toxicity studies still need to be addressed. TMOs can perhaps be exceptional candidates for future biological applications in the oligonucleotide field.

## Figures and Tables

**Table 1:** Solid-phase synthesis parameters used for PMOs and PMO-DNA chimeras.

Reactions	Wash/Reagents/Solvents	Time (Seconds)
Detritylation	10% TMPB + 0.5% TFA in CHCl_3_	Flow 45 s
Condensation	0.1 M Morpholinonucleoside phosphoradiamidite (**15**, **31**, **32**, **33**) in ACN + Activator 0.12 M dicyanoimidazole in ACN	Hold 300 s
	0.1 M Deoxynucleoside phosphoradiamidite (**34**, **35**, **36**, **37**) in ACN + Activator 0.25 M 5-ethylthio-1H-tetrazole in ACN	Hold 180 s
Boronation	0.05 M BH_3_-THF in THF	Flow 15 s, Hold 45 s
Oxidation	0.02 M Iodine in THF/water/pyridine	Flow 8 s, hold 15 s
Capping	Cap A (THF/Pyridine/Ac_2_O) + Cap B (16% N-methylimidazole in THF)	Flow 10 s, hold 5 s

**Table 2:** Solid-phase synthesis cycle used for MOs and MO-DNA chimeras.

Reactions	Wash/Reagents/Solvents	Time (Second)
Detritylation	3% Trichloroacetic acid in Dichloromethane	95 s
Condensation	0.1M 5′-DMTrO-morpholinonucleoside phosphordiamidite in acetonitrile and 0.12 M 5-ethylthio-1H-tetrazole in acetonitrile	600 s
	0.1M 5′-DMTrO-2′-deoxynucleoside phosphoramidites and 0.12 M 5-ethylthio-1H-tetrazole in acetonitrile	30 s
Capping	Cap A (THF/Pyridine/Ac_2_O) + Cap B (16% N-methylimidazole in THF)	5 s
Oxidation	0.1 M (1S)-(+)-(10-camphorsulfonyl)-oxaziridine in anhydrous acetonitrile	180 s

**Table 3:** Solid-phase synthesis parameters used for TMOs and TMO-DNA chimeras.

Reactions	Wash/Reagents/Solvents	Time (Seconds)
Detritylation	3% Trichloroacetic acid in Dichloromethane	Flow 45 s
Condensation	0.1 M Morpholino hosphorodiamidite (**15**, **38**, **39**, **40**) in can + Activator 0.12 M 5-ethylthio-1H-tetrazole canACN	Hold 300 s
	0.1 M 2′-Deoxynucleoside 3′-phosphoradiamidite (**41**, **42**, **43**, **44**) in ACN + Activator 0.12 M 5-ethylthio-1H-tetrazocanin ACN	Hold 30 s
Thiolation	3-[(Dimethylaminomethylene)amino]-3H-1,2,4-dithiazole-5-thione (DDTT)	360 s
Capping	Cap A (THF/Pyridine/Ac_2_O) + Cap B (16% N-methylimidazole in THF)	Hold 5 s

## Data Availability

Not applicable.
